# Glioblastoma Multiforme Therapy and Mechanisms of Resistance

**DOI:** 10.3390/ph6121475

**Published:** 2013-11-25

**Authors:** Yulian P. Ramirez, Jessica L. Weatherbee, Richard T. Wheelhouse, Alonzo H. Ross

**Affiliations:** 1 Department of Biochemistry and Molecular Pharmacology and Department of Cancer Biology, University of Massachusetts Medical School, 364 Plantation Street, Worcester, MA 01605, USA; E-Mails: Yulian.Ramirez@umassmed.edu (Y.P.R.); Jessica.Weatherbee@umassmed.edu (J.L.W.); 2 School of Pharmacy, University of Bradford, Bradford BD7 1DP, UK; E-Mail: r.t.wheelhouse@bradford.ac.uk

**Keywords:** angiogenesis, autophagy, imidazotetrazine, MGMT, DNA repair, temozolomide, cancer stem cells

## Abstract

Glioblastoma multiforme (GBM) is a grade IV brain tumor characterized by a heterogeneous population of cells that are highly infiltrative, angiogenic and resistant to chemotherapy. The current standard of care, comprised of surgical resection followed by radiation and the chemotherapeutic agent temozolomide, only provides patients with a 12–14 month survival period post-diagnosis. Long-term survival for GBM patients remains uncommon as cells with intrinsic or acquired resistance to treatment repopulate the tumor. In this review we will describe the mechanisms of resistance, and how they may be overcome to improve the survival of GBM patients by implementing novel chemotherapy drugs, new drug combinations and new approaches relating to DNA damage, angiogenesis and autophagy.

## 1. Introduction

Glioblastoma multiforme (GBM) is a grade IV brain tumor characterized by a heterogeneous population of cells that are genetically unstable, highly infiltrative, angiogenic, and resistant to chemotherapy [1]. GBM tumors harbor a series of mutations that provide cells with selective growth advantages that promote survival and proliferation in a hostile and hypoxic environment [2]. For example, 30%–40% of GBM tumors have amplification of the epidermal growth factor receptor (EGFR), a tyrosine kinase receptor that activates MAPK and PI3K signaling [3]. In addition, a subset of GBM tumors expresses an EGFRVIII variant in which the extracellular domain of the receptor is lacking, resulting in constitutive activation [4]. Tumor suppressor genes, such as p53, p21, p16, and PTEN are commonly mutated in GBMs, pointing to the highly unstable nature of the cells [5]. GBM tumors are characterized pathologically by the presence of necrotic areas and an aberrant vasculature comprised of glomeroid tufts and hyperproliferative, leaky and unorganized blood vessels [1].

The current standard of care is surgical resection coupled with ionizing radiation (IR) and the chemotherapeutic agent temozolomide (Temodar^®^, Temodal^®^, TMZ) [6,7]. However, this treatment only provides GBM patients with a 12–14 month survival period post-diagnosis [6,7]. Despite aggressive surgical resection and chemotherapy, almost all GBM patients undergo tumor recurrence. Ninety percent of GBM tumors have been shown to recur at the primary site [1]. This can be partly attributed to the highly infiltrative nature of the tumor, making complete resection with clean margins nearly impossible. In addition, GBM tumors can have extensive regions of hypoxia. This reduction in oxygen may limit the efficacy of IR as the generation of DNA-damaging free radicals is decreased [8]. The capacity of GBM chemotherapeutic drugs to cross the blood brain barrier (BBB) and enter the tumor limits efficacy [9,10]. The abnormal and leaky tumor vasculature causes high hydrostatic pressure in the tumor, thereby, reducing drug delivery to the tumor. It was proposed that by placing dissolvable chemotherapy wafers (Gliadel^®^) in the tumor bed, these obstacles would be diminished or overcome [11,12]. However, even with IR, TMZ and Gliadel^®^ combined treatments, GBMs include a population of cells that survive the IR and TMZ treatments and may form a pool of even more chemotherapy-resistant cells.

In the following sections we will address mechanisms of resistance, such as: DNA damage response pathways, cancer stem cells, microenvironment-mediated chemotherapy resistance, tumor-derived endothelial cells, and autophagy and how these mechanisms can be targeted for therapy.

## 2. Glioma Chemotherapy: TMZ and Gliadel^®^

**Scheme 1:**
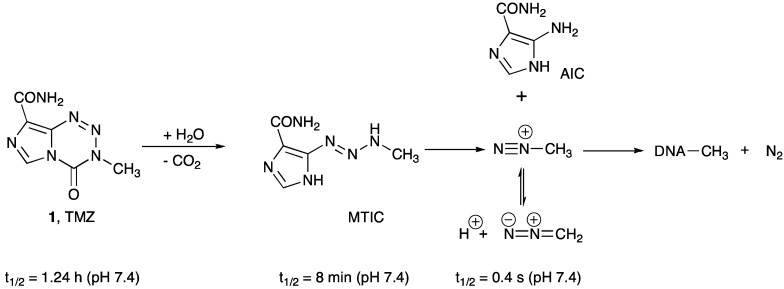
Prodrug activation of temozolomide.

TMZ is an acid-stable orally administered alkylating drug that crosses the BBB [13]. It has excellent uptake and distribution behavior, and there is direct evidence of tumor localization [14]. TMZ is a prodrug, and its aqueous chemistry is typical of imidazotetrazine compounds (Scheme 1). It undergoes hydrolytic ring opening at neutral or alkaline pH under purely chemical control, and the first significant intermediate is the open-chain triazene MTIC [15] (Scheme 1).

**Scheme 2:**
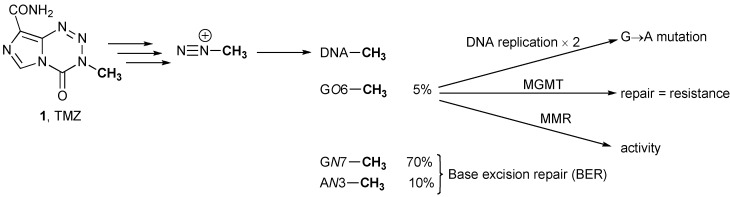
Biological fate of methyldiazonium ions.

The activated intermediate MTIC is shared with dacarbazine, a prodrug used against malignant melanoma, which in contrast, requires hepatic demethylation to release MTIC, from which methyldiazonium is released, which methylates DNA (Scheme 2). The majority (70%) of the methyl groups transferred to DNA appear at *N*7-guanine sites with only about 10% at *N*3-adenine and 5% at O6-guanine [13,16].

**Scheme 3:**
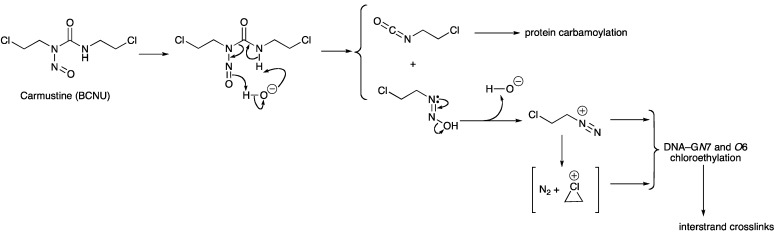
Mechanism of prodrug activation and action of carmustine.

Gliadel^®^ is a biodegradable polifeprosan 20 wafer impregnated with carmustine, a small lipophilic alkylating and interstrand crosslinking nitrosourea [11,12]. There are strong parallels between the mechanisms of prodrug activation and action of carmustine and TMZ (Scheme 3) [17].

Under physiological conditions, spontaneous hydrolysis results in fragmentation of the nitrosourea to release an alkyldiazoinium ion (in this case chloroethyldiazonium) and an isocyanate [18]. Subsequent reaction of the isocyanate with biological macromolecules is not a major contributor to the pharmacology. Chloroethyldiazonium in aqueous systems has a complex fate [19,20], but the therapeutic activity is derived from guanine chloroethylation of DNA, in particular at G-*O*6 positions, and further reaction of the monoalkylation adducts to form interstrand crosslinks.

Gliadel^®^ wafers are implanted in the cranial resection cavity prior to IR treatment. The Gliadel^®^ wafers produce high local concentrations of carmustine directly into the tumor bed after surgery when the tumor burden is low [21,22]. The rationale for this approach is that the resection cavities are relatively avascular and Gliadel^®^ may target cells missed by systemically administered TMZ or carmustine. Furthermore, the wafers release carmustine for several weeks. In contrast, systemically administered carmustine persists only for a few hours. Clinical trials demonstrated that Gliadel^®^ wafers are safe for both newly diagnosed and recurring GBMs [23,24]. IR plus Gliadel^®^ showed greater overall survival (OS) than IR alone. However, the combination of IR, TMZ and Gliadel^®^ did not show a statistically significantly increase in survival over IR and TMZ. As a result, IR and TMZ continue to be the standard therapy for GBMs.

## 3. DNA Damage Repair

### 3.1. Methyl Guanine Methyl Transferase (MGMT)

**Scheme 4:**
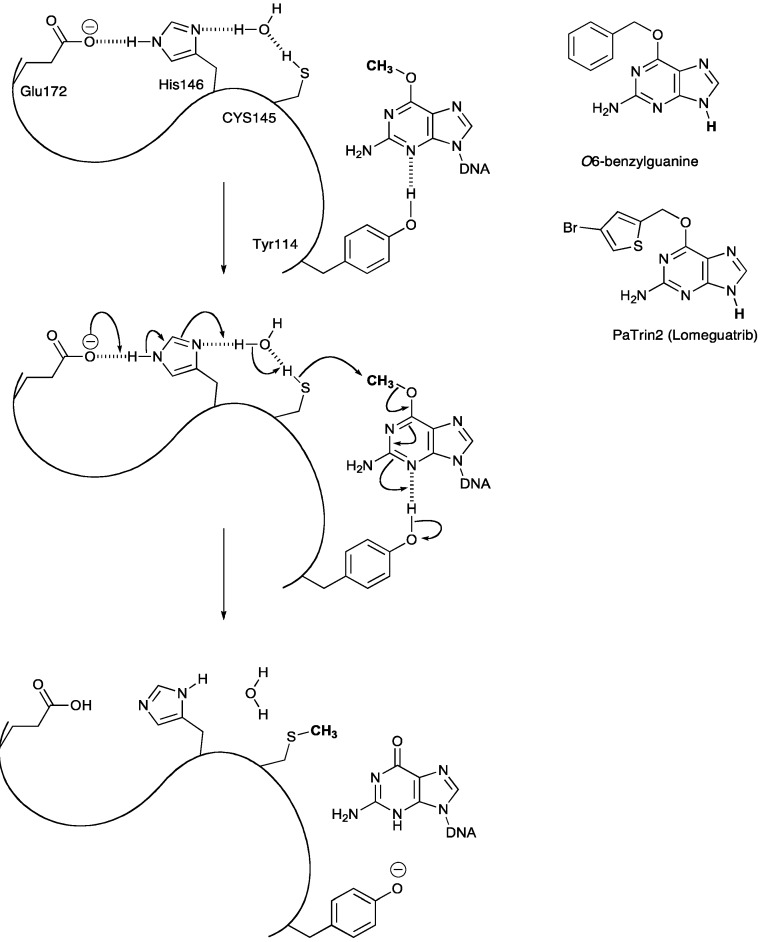
Mechanism of action of MGMT and structures of the two clinically tested MGMT inactivators.

The best-documented mechanism of resistance to TMZ is mediated by the DNA repair protein MGMT, which removes methyl groups from *O6-*MeG lesions that arise from TMZ treatment [25]. During the repair process, the modified base is flipped out of the double helical stack so it can enter the MGMT active site; its position in the DNA duplex being taken by a lysine residue [26]. In the active site, base-catalysis generates a reactive thiolate nucleophile from cysteine 145 (in the human form). This cleaves the C-O bond of *O*6-MeG in a nucleophilic substitution reaction that results in a mixed thioether product, leading to the inactivation of the protein (Scheme 4) [27]. MGMT is thus a reagent consumed stoichiometrically during the repair reaction, not an enzyme. In the context of TMZ antitumor activity, DNA repair by MGMT is the primary mechanism of drug resistance.

MGMT expression inversely correlates with sensitivity to the alkylating agents TMZ and carmustine in glioma cells and glioma stem-like cells [28,29,30]. Differentiated cell lines with elevated levels of MGMT show increased chemoresistance [31,32]. This dependence has been demonstrated by treating glioma, leukemia, ovarian, and breast cell lines with suicide inactivators of MGMT, *O6*-benzylguanine (*O6*-BG) [33] or 6-[(4-bromo-2-thienyl)methoxy]-9*H*-purin-2-amine (PaTrin-2) [34,35,36]; MGMT inhibition increased sensitivity to TMZ treatment.

Methylation of the MGMT promoter occurs in approximately 45% of newly diagnosed glioblastoma patients and is prognostic for response to TMZ treatment [37]. Patients with MGMT promoter methylation have increased survival when treated with radiotherapy in combination with TMZ, while patients with MGMT-positive tumors do not benefit as greatly from this dual treatment [6,28]. Several methods can be employed to determine MGMT status (mRNA levels, protein levels by immunohistochemistry (IHC), promoter methylation, and enzyme activity); however, current evaluations in the clinic usually only assess MGMT protein expression and promoter methylation. It remains unclear which technique has the most prognostic value in the clinical setting.

In a new retrospective study, Lalezari *et al.* [38] focused on 418 patients with newly diagnosed GBMs, of whom 410 were treated with IR and TMZ. Tumors were analyzed for MGMT protein expression via IHC, promoter methylation by methylation-specific PCR (MSP), and individual CpG sites were analyzed by bisulfite sequencing (BiSEQ). Low MGMT protein expression (<30% positive cells) and high promoter methylation individually correlated with OS and progression-free survival (PFS). MGMT MSP correlated with MGMT IHC, and IHC status stratified outcome in the methylated group. This data was further validated by BiSEQ analysis of 24 CpG sites within the differentially-methylated region 2 (DMR2) of the MGMT promoter. Protein levels inversely correlated with methylation density in the DMR2 and showed that hypermethylation (≥3 CpG sites) was correlated with higher OS and PFS. Combining analyses of protein expression and promoter methylation offers superior prognosis than individual analyses of these factors and was recommended for testing of newly diagnosed GBMs [38].

A subpopulation of glioblastoma patients have low MGMT expression with no detectable promoter methylation [25], indicating that other molecular mechanisms also regulate MGMT expression. Recently, Kreth *et al.* [39] focused on the post-transcriptional regulation of MGMT and found that MGMT was subject to alternative polyadenylation, giving rise to transcripts with varying 3′UTR. The longer 3′UTR was expressed in tumors and absent in normal brain tissue. MGMT protein levels were reduced when the elongated transcript was expressed. These results were independent of the promoter methylation and were attributed to decreased mRNA stability as a result of miRNA regulation. This study provides an explanation for tumors with unmethylated MGMT promoter and low MGMT expression and provides further insight into molecular mechanisms that regulate MGMT expression. Further studies are needed to evaluate whether the long 3′UTR MGMT transcript is a prognostic factor for survival of GBM patients.

### 3.2. MGMT Therapeutic Targets

Inhibition of MGMT in combination with TMZ has been studied as an approach to improve treatment of GBMs in the clinic. *O6*-BG is a small-molecule pseudosubstrate that transfers a benzyl group to the MGMT active site cysteine 145 residue, thereby, inactivating MGMT and preventing removal of methyl groups from the DNA [26]. Initial phase I clinical trials showed that *O6*-BG effectively inhibits MGMT in GBM tumors, but TMZ therapy in combination with *O6*-BG was limited by myelosuppression [40]. This enhanced toxicity is attributed to *O6*-BG inhibition of the low levels of MGMT in hematopoietic progenitor cells. Studies on MGMT-/- mice, also demonstrated that damage to bone marrow was the main source of toxicity. This effect can be averted by transplantation of wild-type bone marrow into MGMT-/- mice [41]. A new clinical trial will explore the feasibility of infusing hematopoietic progenitors modified to express MGMT via a retroviral vector as a way to overcome the limitation of therapy-induced myelosuppression [42]. 

One therapeutic strategy that has been evaluated is the use of increased doses and prolonged scheduling of TMZ as a means of depleting MGMT. This approach was shown to decrease MGMT activity in peripheral blood mononuclear cells [43]. In addition, a recent phase II study of 58 patients, with first recurrences, evaluated the efficacy and safety of a 21 days on/7 days off regimen at 75–100 mg/m^2^/day. This study only included patients who had previously received TMZ concomitant and adjuvant IR, and the study was ended when second progressions occurred. This regimen proved to be safe, but none of the patients achieved a complete response. Partial responses for 13% of patients were observed as well as 6-month PFS of 11%, showing this regimen had little efficacy for recurrent tumors [44].

Other approaches utilize RNAi to directly interfere with MGMT protein expression. Using MGMT-siRNAs and a novel liposome, LipoTrust™ EX Oligo for delivery, MGMT was efficiently knocked down in glioma cells lines, GBM-stem like cells, and *in vivo* glioma tumors. *In vivo* delivery was effective whether administered intratumorally in a subcutaneous model or via an osmotic pump in an intracranial model. Both *in vitro* and *in vivo*, MGMT siRNA enhanced sensitivity to TMZ [45]. Another RNAi approach employed a lentiviral-based technology to target MGMT with a small hairpin RNA [46]. MGMT was successfully inhibited in TMZ-resistant glioma cultures, enhancing sensitivity to TMZ for tumors implanted into the flanks of nude mice. Efficient *in vivo* transduction of the shMGMT vector into GBM xenografts decreased MGMT expression and inhibited tumor growth following TMZ treatment. Although this seems a promising therapy, the efficacy and toxicity of these viral vectors require further evaluation.

Post-translational regulation of MGMT occurs by the 26S proteasome, making this a candidate for therapy. Bortezomib (BTZ, PS-341) is a boronic acid dipeptide that inhibits the proteasome and markedly reduces levels of MGMT mRNA and protein [47]. Efficacy of combined BTZ and TMZ therapy differed between glioma lines and was schedule-dependent. MGMT-negative U87MG cell line showed decreased viability and increased apoptosis when TMZ was administered before BTZ, while the opposite was true for MGMT-positive T98G cells. This effect was partially mediated through MGMT downregulation [47] and speaks to the importance of sequence of therapy in combination treatments. Primary glioma stem-like cells were more sensitive to proteasome inhibition by BTZ than normal neural stem and progenitor cells due in part to the lower proteasome activity [48], making it an attractive therapy to combat recurrence. Phase I studies showed BTZ to be well tolerated with thrombocytopenia being the most common toxicity [49,50]. BTZ is now clinically approved for hematopoietic malignancies [50].

### 3.3. Mismatch Repair (MMR)

The responses to TMZ treatment do not absolutely correlate with MGMT, leading us to believe that additional mechanisms are at play. One mechanism thought to mediate resistance is loss of MMR [51]. *O6-*MeG lesions mismatched with thymine bases are recognized by the MMR. The thymine residue is excised; however, in the absence of MGMT, the *O6-*MeG remains, and, thymine is reinserted opposite the *O6-*MeG. These futile cycles of repair result in activation of ATR and Chk1, generation of double-strand breaks (DSBs) and eventually apoptotic cell death [13]. Cells with MMR deficiencies do not process the mismatch, DNA replication proceeds, and no cell cycle arrest or apoptosis occurs. The triggering of cell cycle arrest is FANCD2-dependent, but not ATR-dependent [52]; this response is more reminiscent of a DNA crosslinker than a monoalkylator [51].

Many groups have examined the role that MMR plays in mediating responses in the clinic to TMZ with conflicting results. In one study, 52 glioma patient samples were assessed for microsatellite instability (MSI), which is thought to be a result of MMR gene inactivation [53]. Zero patients exhibited high MSI, defined as instability in three of five loci analyzed. Direct sequencing of MSH6 identified mutations, many of which did not hinder generation of wild-type protein. In this study MMR deficiency does not appear to contribute to resistance to TMZ therapy [54]. A low MSI rate of 8.5% was found in a larger panel of 129 GBM patients and a higher presence of MSI was detected amongst the 20 GBMs that had recurred. Consistent with the previous studies, no high MSI was detected, and IHC for MMR proteins showed aberrant expression in only one tumor with MSI [53]. A larger scale analysis of 624 gliomas further validated the lack of high MSI with an incidence of 0.16% [55]. Paired analysis of primary and recurrent tumors, noted no differences in PMS2, MLH1, MSH2, and MSH6 expression, and promoters of these genes remained unmethylated in both instances [25]. Similarly another study saw no apparent correlation between MSH2, MSH6, and PMS2 protein and sensitivity to TMZ [28]. Single nucleotide polymorphism (SNPs) analysis of patient samples treated with radiation alone or with TMZ showed that 50% harbored MSH6 G268A polymorphisms. However, no OS benefit was noted between samples harboring or lacking MSH6 G268A [56]. 

In contrast, Rellecke *et al.* [57] observed that all primary *de novo* glioma cultures in their study had detectable transcripts and proteins for MMR genes except for MSH2, which they stratified into high and low expression levels. The chemosensitivity of these cells to a panel of chemotherapeutic agents, including carmustine, cisplatin, and taxol, was evaluated with 36% of the cultures showing insensitivity to all of the agents tested. These cultures were characterized by high expression of MSH2, which is thought to be a source of resistance in these cells [57]. Yip *et al*. [58] focused their studies on a cohort of The Cancer Genome Atlas (TCGA) recurrent tumors, which had been previously found to have MSH6 mutations. Analysis of samples pre and post exposure to alkylating agents showed the MSH6 mutations were not present in pre-treatment samples indicative that these mutations arose as a result of therapy. This same observation carried over to *in vitro* work with an A172 glioma line selected to be resistant to TMZ. The TMZ resistant line, A172TR3, had reduced sensitivity to TMZ, decreased expression of MSH6 and a MSH6 somatic mutation. Similarly, knockdown of MSH6 in the glioma U251 line reduced sensitivity to TMZ. All these results were independent of MGMT as the glioma lines tested were negative for MGMT as well as the TCGA recurrent samples. However, in agreement with previous studies high MSI was not detected [58]. Some of the contradictory reports may be attributed to the fact that high levels of MSI have been correlated to deficient MMR and thus used as a readout for MMR deficiency, despite reports indicating no correlation between the two [54,58]. One hypothesis is that the low levels of MSI observed in some cases might be a result of minor MMR players, which are not tested in these analyses [53].

Despite the complex interpretation of MSI, we conclude that both the MGMT and MMR pathways play major roles in the tumor response to TMZ treatment. A tumor low in MGMT will respond well to initial TMZ therapy but at the cost of accumulated mutations. Surviving tumor cells are likely to have acquired MMR mutations, resulting in acquired tolerance to further TMZ therapy: a situation typical of GBMs in the clinic.

## 4. Targeting a Complex Vasculature: GBM Cancer Stem Cell, GBM Endothelial Cells, and Angiogenic Resistant Mechanisms

### 4.1. Rationale for Targeting GBM Vasculature

Judah Folkman proposed in 1971 that inhibition of angiogenesis, the process whereby new blood vessels are generated by the proliferation of pre-existing ones, would be an effective anti-tumor therapy [59]. Like normal tissues, tumors require a vascular network to deliver nutrients and oxygen, and remove harmful metabolic waste products. Tissues exceeding more than 70 μm from blood vessels are prone to hypoxia, which if not resolved, leads to apoptosis [8]. As GBM tumors have a highly proliferative, albeit abnormal vasculature, it seemed plausible that inhibition of angiogenesis would reduce tumor growth and improve the survival of GBM patients. However, despite promising *in vitro* data, the implementations of anti-angiogenic drugs have been challenging as GBM tumors adapt and become resistant to therapy. Potential mechanisms will be discussed in further detail below but include resistant GBM cancer stem cells (CSCs), differentiation of CSCs into glioblastoma-derived endothelial cells (GECs), increased invasion of hypoxic cells, and activation of alternative angiogenic pathways.

### 4.2. Identification of Plastic Neural Stem Cells in the Adult Brain

In 1992, Reynolds and Weiss isolated a population of cells from the striatum of adult mice that could be maintained in a non-differentiated self-renewing state but differentiate into astrocytes and neurons when cultured on adherent plates [60]. Okano *et al.* expanded this work by finding that adult neural stem cells could regenerate and form functional neurons to replace damaged or lost ones [61]. These findings revolutionized neurobiology suggesting that a population of neural stem cells could be maintained throughout adulthood, refuting the long-held 1928 proposal [61,62] that neurogenesis only occurs during embryonic and early post-natal development, and that damaged neuronal cells cannot be replaced in the adult brain. 

In 2004 Wurmser *et al.* [63] showed that GFP^+^ murine neural stem cells cultured with human endothelial cells gave rise to GFP^+^ endothelial cells, suggesting that neural stem cells differentiate into endothelial cells. Researchers observed that neural stem cells localize around blood vessels [64,65,66], suggesting an interaction between stem and endothelial cells. This differentiation was not due to cell fusion as the GFP^+^ endothelial cells displayed a normal karyotype but was dependent upon cell-cell contact of neural stem cells with endothelial cells. In culture, these GFP^+^ endothelial cells retained endothelial cell markers and formed tubules, the functional capillaries formed by endothelial cells. These data confirmed that adult neural stem cells are plastic and provided a novel mechanism of angiogenesis in the adult brain.

The pioneering work of Reynolds and Weiss [60] and Wurmser *et al.* [63] laid the foundation for: (a) the development of the cancer stem cell hypothesis and (b) how stem cells can contribute to brain vascularization—two important fields that are essential to understanding the development, maintenance, and resistance of GBM tumor cells.

### 4.3. GBM Cancer Stem Cells Differentiate into Glioblastoma-Derived Endothelial Cells

GBM is characterized by an aberrant vasculature comprised of hyper-proliferative endothelial cells, glomeroid tufts, and disorganized blood vessels—phenotypes that are absent in lower grade brain tumors [67]. The reason for this characteristically aberrant vasculature in GBMs has remained elusive for decades; however, recent findings have begun to elucidate explanations for this aberrant vasculature.

Starting in 2010, several groups published findings suggesting that endothelial cells contain the same genetic aberrancies found in GBM tumors cells [68,69,70,71]. Ricci-Vitiani *et al.* [68] observed p53 mutated endothelial cells lining the lumen of blood vessels in GBM archived material. Wang *et al.* [69] found endothelial cells with amplified chromosome 7, an amplification characteristic of GBM tumor cells that results in over expression of EGFR. These observations led to the hypothesis that these mutated endothelial cells are derived from GBM CSCs, consistent with the precedent that neural stem cells can differentiate into endothelial cells [63]. To determine if CSCs can differentiate into GECs, Wang *et al.* [69] isolated a population of cells from human GBM tumors that co-expressed the stem cell marker, CD133^+^, and the endothelial progenitor marker, CD144^+^. Culture of these double positive cells in endothelial rich media decreased expression of the CD133^+^/CD144^+^ markers and increased markers for mature, proliferating endothelial cells. Furthermore, the differentiated endothelial cells derived from the CSCs showed uptake of acetylated DiI-low density lipoprotein, an assay used to mimic the functional capability of endothelial cells to transport fluids, suggesting that GECs are functional. It should be noted that not all GECs were functional and a portion of them formed structures reminiscent of glomeroid tufts when plated on Matrigel, indicating that GECs may contribute to the abnormal vasculature of GBMs. Furthermore, *in vivo* lineage tracing of GFP^+^/CD133^+^ cells implanted into nude mice resulted in GFP^+^/CD105^+^ cells negative for murine endothelial markers, indicating that the *in vivo* differentiation of a CSC to a rapidly proliferating GBM endothelial cell is possible. However, a recent study reported GBM tumors are comprised of a low percent of tumor-derived endothelial cells (TDECs) which were not found to be incorporated in the blood vessel. This group questions the clinical relevance of TDECs [72].

This exciting discovery led researchers to question whether GECs affect tumor angiogenesis and if it is possible to target the pathways that regulate GEC differentiation to reduce tumor vasculature development. Ricci-Vitiani *et al.* [68] determined that suppressing differentiation of GBM neurospheres to endothelial cells reduced tumor growth and eliminated vascular glomeruli, suggesting that GECs contribute to GBM tumor growth and vasculature development. Wang *et al.* [69] demonstrated that both NOTCH, which is essential for the maintenance of CSCs, and VEGF pathways regulate the differentiation of GBM cancer stem cells to GECs. Treatment of CD133^+^ cells with bevacizumab (Avastin^®^), a monoclonal antibody against VEGFA, did not block progression of CD133^+^ cells to an early endothelial state (CD133^+^/CD144^+^), but did block double positive cells from differentiating into CD105^+^ cells, suggesting that VEGFA is essential for double positive cells to reach a mature, rapidly proliferating endothelial state. Conversely, when CD133^+^ cells were treated with the small molecule NOTCH inhibitor, DAPT, the CD133^+^ cells were unable to transition into early endothelial cells (CD133^+^/CD144^+^); however, DAPT treatment did not block the differentiation of double positive cells from maturing into CD105^+^ cells. CD105 is a marker for endothelial progenitor cells and is absent from normal adult brain. This suggests that VEGFA is necessary for double positive cells to reach a mature endothelial cell state while inhibition of NOTCH signaling blocks cells from differentiating into endothelial progenitors.

In contrast, Soda *et al.* [70] suggest that differentiation of murine CSCs to GECs is regulated by hypoxia and is VEGF independent. The group found that only a small population of murine TDECs express VEGFR2 and, although the cells do secrete VEGF, receptor or pathway inhibition does not prevent the formation of tubules *in vitro*. Most noteworthy was the observation that no significant increase in survival resulted when tumor-bearing mice were treated with vehicle *versus* VEGFR inhibitor. Surprisingly, the VEGFR-inhibited mice had a statistically significant increase in TDECs *versus* vehicle treated mice, suggesting that the TDECs are resistant to VEGF inhibition. This could explain in part why bevacizumab has only a transient effect in the clinic. It should be noted that Soda *et al.* [70] analyzed TDECs generated by a GFAP-Cre/p53 heterozygous mouse injected with Cre-dependent lentiviruses bearing oncogenes H-Ras and Akt while the aforementioned groups studied GBM endothelial cells isolated from human tumors [68,69]. It is possible that the discrepancies between the groups regarding VEGF dependence is due to differences in the models and markers used to analyze GBM CSC to endothelial differentiation.

A recent study suggests that TDECs may also contribute to IR resistance [73]. When GBM cells were differentiated to an endothelial cell-like lineage, these cells had decreased apoptosis but increased senescence, indicating that the surviving cells are resistant to treatment.

Lastly, recent data suggests that GBM stem cells further contribute to tumor vascularization by differentiating into pericytes, the cells that wrap around endothelial cells to support and maintain them [74,75]. Reduced pericyte coverage results in a less protected and more exposed blood vessel, increasing the sensitivity of tumor ECs to radiation and chemotherapy. It is beneficial for the tumor if GBM CSCs differentiate into protective pericytes to decrease sensitivity to chemotherapy.

The identification of a population of CSCs that differentiate into endothelial cells harboring the same genetic aberrancies of GBM tumor cells may not only explain the abnormal vasculature observed in GBMs but may also play a role in resistance to anti-angiogenic therapies and IR. The existence of GECs in GBMs may provide a novel therapeutic target in which the inhibition of differentiation may reduce tumor burden via decreased tumor angiogenesis. However, the feasibility and success of the treatment remains to be seen as GBM patients have a moderate and transient response to anti-angiogenic inhibitors. The most efficacious treatments may occur as combination therapies in which anti-angiogenic inhibitors normalizing the leaky GBM vasculature and generate a small window of time for the delivery of chemotherapy agents.

### 4.4. GBM Cancer Stem Cells and Their Microenvironment Contribute to a Chemotherapeutic Resistant Tumor

It has been long noted that GBMs are comprised of a heterogeneous population of cells, and it was assumed that this heterogeneity arose from differentiated cells acquiring mutations or perhaps mutated de-differentiating neural cells. However, several groups in the early 2000s proposed a novel reason not only for tumor heterogeneity but also for tumor initiation [76,77,78]. A small population of cells isolated from GBM tumors lacked differentiated neural markers, had the capacity to self-renew, proliferate, differentiate, and also gave rise to tumors that could be serially maintained while phenotypically mimicking the parental GBM tumor [66]. Researchers suggested that this tumor initiating population of cells were comprised of GBM CSCs. This hypothesis had precedents; it had already been suggested that CSCs initiate non-solid tumors, such as leukemias, and also some solid tumors, such as breast and colon cancers [79]. Although there has been much debate about which markers can be used to isolate CSCs [80], and whether non-CSCs also can initiate tumors [81,82], multiple groups have substantiated the cancer-stem-cell hypothesis [83,84]. A particularly exciting topic for future study is how stem cells affect the tumor microenvironment and promote chemotherapeutic resistance.

In order for a tumor to grow, the vasculature must provide the proliferating tumor cells with oxygen, nutrients, and a means to dispose of toxic metabolic wastes [85]. If a tumor cell is more than 70 μm from a blood vessel, it lacks sufficient oxygen and nutrients, and as a result, experiences a hypoxic environment. To alleviate this hypoxia, cells secrete vascular endothelial growth factor (VEGF), an angiogenic factor that promotes the recruitment, migration, proliferation, and eventually formation of additional blood vessels. GBMs are characterized by a hyperproliferative vasculature, comprised of glomeroid tufts and highly branched but dead-end blood vessels [1]. This aberrant vasculature may be due in part to GBM CSCs themselves secreting VEGF and stromal derived factor 1 (SDF1), thereby, promoting tumor vasculature development [86,87]. In a rat glioma model, C6 cancer stem cells showed increased expression of VEGF and SDF1 *versus* non-CSCs, suggesting that these cells can initiate angiogenesis, the formation of new blood vessel from pre-existing ones and SDF1-mediated vasculogenesis, the *de novo* formation of blood vessels, by recruiting endothelial progenitor cells to the tumor bed [87]. Tumors initiated by C6 CSCs had increased microvessel density, increased proliferation, and more circulating endothelial progenitor cells than non-CSCs, suggesting these cells significantly contribute to the development of tumor vasculature [87]. Rats treated with either a monoclonal antibody that binds VEGFA or a small molecule inhibitor of SDF1, resulted in C6 tumors with reduced vasculature [87]. However, disrupting tumor vasculature with anti-angiogenic inhibitors does not result in complete abolition of CSCs in the vascular niche, and a subset of resilient cells can form.

Although not entirely functional, this vasculature provides tumor cells with nutrients and an aberrant microvascular niche for GBM CSCs. Calabrese *et al.* [65] found that human GBM CSCs (nestin^+^/CD133^+^) preferentially associate with endothelial cells *in vivo*. This interaction was verified *in vitro* as human GBM CD133^+^ cells, when cultured with primary human endothelial cells (PHECs), line the PHEC tubule structures. When CD133^+^ cells and PHECs were cultured in a transwell system, the cells grew five times faster over a two-week span than CD133^+^ cells cultured without PHECs indicating that endothelial cells secrete factors for the maintenance and survival of GBM CSCs and may be essential for the stem-like state. However, another study suggested that a direct physical interaction must occur in order for endothelial cells to maintain the CSC phenotype [88]. Regardless, the data indicates that the two cell types influence each other and this interaction is important for the maintenance of stem cells. This relationship was further substantiated when tumor cells injected intracranially with PHECs formed tumors more rapidly than tumor cells alone, suggesting that PHECs promote CD133^+^ initiated tumor growth [65].

In addition to maintaining CSCs, the microvasculature may serve as a protective niche for CSCs by shielding them from IR and chemotherapeutic agents, such as TMZ. A recent study by Borovski *et al.* [73,88] found that tumor microvascular endothelial cells (tMVECs) isolated from human GBM tumors promoted the proliferation of human CD133^+^ cells when the co-cultures were exposed to IR. Furthermore, tMVECs significantly increased the number of CD133^+^ cells after IR, suggesting they not only promote proliferation but also maintain the CSC population after therapy. In addition to protecting CD133^+^ cells from IR, tMVECs also promoted the proliferation of TMZ-sensitive GBM cultures, indicating that tMVECs protect CD133^+^ cells from chemotherapy. When co-cultured cells (CD133^+^ with tMVECs) were treated with both IR and TMZ, CD133^+^ cells showed increased proliferation, indicating resistance to the standard of care. Different primary GBM lines exhibited different levels of “tMVEC protection,” with varying degrees of re-entry into cell cycle and proliferation. Borovski *et al.* [73,88] found that when tMVECs were treated with IR, a small percentage of cells underwent apoptosis; however, the majority of the cells survived and entered a G2 arrest. The IR-treated cells entered a protective but metabolically active senescent state capable of promoting proliferation and maintenance of CD133^+^ cells. Chemotherapeutic resistant tMVECs were shown to be clinically relevant as post mortem biopsies of GBM patients revealed senescent tumor endothelial cells [73]. These data suggests that tMVECs are inherently resistant to IR-mediated apoptosis and create a protective niche for CSCs. Although the mechanism of this tMVEC-induced protection has not been elucidated, Borovski *et al.* [73,88] suggested that tMVECs may regulate MGMT expression in tumor cells, and increase the response of DNA repair pathways, as well as physically shielding the cancer stem cells from chemotherapy.

Additional resistance of GBM CSCs to chemotherapy may be gained by increased activation of DNA cell cycle checkpoints and repair pathways in CD133^+^ cells. Bao *et al.* [89] found that IR increases the percentage of CD133^+^ cells, and that IR-treated CD133^+^ cells have a four to five fold reduction in early apoptosis *versus* IR-treated CD133^−^ cells. Furthermore, Bao *et al.* [89] observed that IR CD133^+^ cells are capable of generating tumors when intracranially implanted into mice, suggesting that CSCs are resistant to IR and capable of re-populating the tumor after chemotherapy. Enhanced resistance of CD133^+^ cells may be due to increased activation of DNA damage checkpoint proteins, such as ataxia-telangiectasia (ATM) and Chk1 and 2. Activation of these checkpoints results in cell cycle arrest, allowing the CSCs time to repair IR initiated DNA damage. Once the repair is complete, the cell can re-enter the cell cycle and initiate secondary tumors. This was substantiated when Bao *et al.* [89] found that IR CD133^+^ cells can form secondary tumors at similar rates of non-IR-treated CD133^+^ cells, suggesting that IR-treated CD133^+^ cells serve as a source for tumor recurrence. CD133^+^ resistant cells can be sensitized to IR *in vitro* if treated with checkpoint kinase inhibitors, presenting a potential therapeutic target. However, although inhibition of Chk1 or Chk2 is feasible, the clinical application may be limited as non-cancer cells also rely on these pathways to repair DNA damage.

A study by Facchino *et al.* [90] noted the role of increased DNA damage response pathways in mediating CSC IR resistance. It was found that BMI1, a member of the polycomb group that represses gene expression, is enriched in CD133^+^ GBM CSCs, possibly increasing recognition and repair of IR induced DSBs. Partial knockdown of BMI1 delayed repair of DSBs, which resulted in a S phase block as well as increased cell death in IR-treated CD133^+^ cells, suggesting that BMI1 may play a role in promoting CD133^+^ radiation-resistance. In addition to increasing cell-cycle checkpoints, GBM cancer cells may also acquire resistance to IR through the NOTCH pathway. Although the role of NOTCH in maintaining neural stem cell self-renewal and inhibiting neural stem cell differentiation has been well-established, it was not until recently that Wang *et al.* [91] suggested an additional role for this key developmental pathway. Analysis of primary human GBM tumors suggests that the NOTCH1 receptor and the NOTCH1 intracellular domain (NICD), which translocates to the nucleus and drives gene expression, are over-expressed. When CD133^+^ cells isolated from human GBM tumors are IR treated, NOTCH transcription and NOTCH target gene expression is increased. Knockdown of the NOTCH1 receptor in GBM cell lines decreases proliferation and inhibition of NICD in IR CD133^+^ cells significantly decreases clonogenicity, while increasing apoptosis, suggesting that NOTCH protects CD133^+^ cells from IR [92]. CD133^+^ cells engineered to constitutively express NICD2 show increased phosphorylation of AKT, a player in the PI3K pathway that promotes cell survival, and decreased apoptosis and increased clonogenicity in response to IR. This finding corroborates a previous study that GBM tumor cells require AKT activation to survive [93]. Interestingly, neither decreased clonogencity nor increased apoptosis was observed in CD133^−^ cells treated with a NOTCH inhibitor and then irradiated, suggesting that NOTCH may preferentially protect CD133^+^ cells [91]. This may occur because CD133^−^ cells are more differentiated and do not rely on NOTCH to maintain stem cell-like behavior.

Furthermore, therapies, such as IR and TMZ, primarily induce apoptosis in rapidly proliferating cells; however, Chen *et al.* [94] suggest that CSCs are relatively quiescent. Using a conditional mouse model in which CSCs are GFP^+^, Chen et al [94] found that after treatment with TMZ, it is the CSC GFP^+^ cells that give to secondary tumor formation, suggesting that CSCs are resistant to current chemotherapeutics not only by up-regulating genes that promote survival, but also by their inherently slow cycling nature.

In summary, GBM CSCs reside in microvascular niches that promote stem cell maintenance and protect the population from chemotherapy. CSCs promote vasculogenesis as well as angiogenesis, can become resistant to chemotherapy by up-regulating cell cycle checkpoints and survival pathways, and may mediate tumor recurrence. In addition, recent data suggest that CSCs can differentiate into TDECs, providing GBMs with an inherent source of tumor vasculature.

### 4.5. Bevacizumab: A Story of Success and Failure

Glioblastoma tumors are characterized by increased VEGF expression as tumor cells secrete this key angiogenic factor [86,87]. Over-expression of VEGFA activates the VEGFR pathway, promoting the proliferation, migration, and survival of endothelial cells, resulting in the formation of tumor blood vessels. As previously mentioned, tumor angiogenesis is further aided by CSCs differentiating into GECs [68,69,70,71]. This creates an aberrant vasculature niche that provides tumor cells with the ability to survive in an otherwise hypoxic and hostile environment. Researchers are currently pursuing compounds that normalize tumor vasculature to disrupt tumor growth and enhance drug delivery [95].

The FDA approved the first human monoclonal antibody against VEGFA, bevacizumab, as a second-line treatment for patients with recurrent GBMs [10,67,96]. The rationale is that antibody-bound VEGF is unable to interact and activate the VEGFR 1 and 2 pathways, resulting in decreased tumor vasculature formation [10,97]. Bevacizumab treatment was proposed to decrease angiogenesis, edema, and tumor burden in GBM patients [98]. A phase II study found that recurrent GBM patients experienced an increased six-month progression-free survival from 9%–15% to 25% when treated with bevacizumab (15 mg/kg, every three weeks) and had an overall six-month survival of 54% [97]. A second clinical trial suggested that if recurrent GBM patients are treated with bevacizumab at a lower dose but at higher frequency (10 mg/kg, every 2 weeks), the estimated six-month PFS can be increased from 25% to 42.6% [67,97]. Second time relapsed GBM patients had a decreased six-month PFS when treated with bevacizumab (27.8% *versus* 42.6%), suggesting that GBM tumors cells become resistant to the antibody and activate alternative angiogenic pathways that are VEGF independent [10]. Analysis of GBM tumor tissues suggests that increased ligand to receptor ratio of VEGFA to VEGFR2 correlates negatively with survival; however, this correlation was not statistically significant [97]. As the study was comprised of a small number of patients, the results need to be verified in a larger patient cohort.

Researchers have proposed using bevacizumab in combination with known chemotherapeutics [10,67,99]. It was suggested that bevacizumab temporarily normalizes the hyper-proliferative and leaky vasculature of GBM tumors, thereby, enhancing delivery of secondary chemotherapeutic drugs. The combination of bevacizumab with irinotecan (Camptosar^®^), a topoisomerase I inhibitor, was suggested as a potential treatment for recurrent GBM patients for three reasons [10]. First, the combination of bevacizumab with irinotecan is efficacious in other aggressive solid tumors; for example, bevacizumab plus irinotecan increased the OS of metastatic colorectal cancer patients *versus* single agent or placebo. Second, irinotecan crosses the BBB, making it relevant for the treatment of GBM patients. Third, a phase II study found that 15% of recurrent GBM patients had a partial response to irinotecan as a single agent. One study suggests that combination of bevacizumab (10 mg/kg) with irinotecan (either 340 mg/m^2^ or 125 mg/m^2^) results in increased six-month PFS from 42.6% with bevacizumab alone to 50.3% with bevacizumab plus irinotecan [67]. A second phase II study with twenty-three grade IV recurrent GBMs found that this combination induced thirteen partial responses [10]. Combination therapy suggests an improvement over single agent alone as bevacizumab, when given as a single agent at 15 mg/kg every three weeks, resulted in a median OS of 6.5 months [97] while combination treatment (bevacizumab 10 mg/kg; irinotecan either 340 mg/m^2^ or 125 mg/m^2^) resulted in a 40 week (about 10 month) OS [10]. However, some GBM tumors do not decrease in size, and these GBM patients have a two and a half month median survival [97]. Thus, targeting tumor vasculature, whether by single or combination therapy, has been challenging for glioblastomas.

To understand the mechanisms that promote bevacizumab resistance, researchers analyzed tumors from three GBM patients that initially responded but then relapsed [100]. Prior to treatment, the initial tumor biopsies contained abnormal and increased vascular proliferation. After bevacizumab treatment, the tumors had almost no hyper-proliferative blood vessels, glomeroid tufts, or proliferating endothelial cells. The relapses seemed counter-intuitive as the data suggested that the patients were responding to the treatment. However, MRIs of the GBM patients indicated that the bevacizumab-resistant tumors were highly infiltrative following treatment. IHC of the resistant tumors suggested potential mechanisms for this increased invasiveness as increased hypoxia and levels of insulin binding protein 2 and matrix metalloproteinase 2 were found.

Researchers began to explore the paradox of bevacizumab-induced tumor invasiveness using immunodeficient mice to recapitulate the observations in GBM patients [100]. Researchers intracranially injected a non-invasive GBM cell line into mice, which were treated for four to six weeks with bevacizumab. They observed that a subset of tumors became highly invasive in response to the treatment and determined via IHC analysis that these tumors had decreased vascular proliferation but increased expression of MMP2, consistent with the bevacizumab-resistant human GBM tumors [100]. To delineate the mechanism driving increased infiltration, one group subcutaneously implanted a GBM cell line, U87, into the flanks of mice and treated them with bevacizumab every three days for 40 days, creating bevacizumab-resistant tumors [101]. Tumor samples were collected over the time course, allowing analysis of the molecular changes in these tumors, which, by day 40, were resistant to bevacizumab. IHC of resistant tumors found that CD31 and CD34, well-established endothelial cell markers, were decreased, blood vessel density was reduced, and the tumors expressed elevated levels of HIF1 alpha. This suggested that bevacizumab reduced the tumor vasculature but as a result, created a hypoxic environment. Microarray analysis revealed that genes regulating glycolysis were up-regulated while genes regulating oxidative respiration were down regulated in bevacizumab-resistant *versus* sensitive tumors, suggesting that bevacizumab treatment induces a shift from mitochondrial respiration to glycolysis, a possible mechanism of resistance [101,102]. The microarrays also indicated that HIF targets were up-regulated, such as the glucose transporter Glut1, and key players in the TCA cycle, succinate dehydrogenase and fumarate, which also act as tumor suppressor genes, were down-regulated. Researchers then proposed that drugs that inhibit glycolysis may increase the efficaciousness of bevacizumab as GBM cells are forced to used oxidative respiration [101,102]. When mice were treated with both bevacizumab and dichloroacetate (DCA), a known inhibitor of glycolysis that can cross the BBB, the combination treatment significantly decreased tumor growth *versus* either agent alone [101]. Combination-treated tumors had decreased Ki67 staining, suggesting that the dual treatment resulted in decreased proliferation that was cytostatic with no significant changes in necrosis between single or double treated tumors. The reduced tumor growth observed in the combined therapy may be due to bevacizumab decreasing tumor vasculature, thereby creating a hostile, hypoxic environment for the GBM cells, which is exacerbated by DCA. In a small study, the combination of DCA with the standard of care showed some tumor regressions [103]. This suggests that DCA may have some efficacy when combined with the standard of care and perhaps may have increased efficacy when used in combination with bevacizumab.

The development of drugs that inhibit the formation of GBM vasculature, reduce tumor growth, and extend the OS of patients is limited and now associated with a switch to invasion and metastasis. The most promising of anti-angiogenic drug, bevacizumab, has shown some success in reducing GBM tumor burden and normalization of the vasculature; however, it is linked to increased tumor invasiveness [100,102]. In addition, GBM patients become less sensitive to the treatment over time. This resistance could be due in part to the fact that the monoclonal antibody only targets one member, VEGFA, of the five members of the VEGF family, allowing other VEGFs to compensate [10]. Resistance can also occur by activating other angiogenic pathways. For instance, EGFR, which is amplified and over-expressed in GBM tumors, can contribute to angiogenesis as well as the well-studied NOTCH/Dll4 interaction and ANG/Tie pathway [10,104,105,106]. Despite great progress, much remains to be resolved in order to develop successful anti-angiogenesis therapies to extend the OS of GBM patients.

## 5. Autophagy

Macroautophagy, referred to as autophagy here, is the process by which cells degrade and recycle cellular content in response to stress or starvation providing the cell with a source of energy until nutrients become available. During this process, a double-membrane cytosolic vesicle, known as the autophagosome, envelopes macromolecules and even whole organelles. Autophagosomes fuse with lysosomes to form autolysosomes, resulting in the degradation of cellular contents. Autophagy occurs in cells at a basal level and is required for homeostasis (as reviewed by [107,108,109]). In the context of glioma cells, autophagy acts as a mechanism following chemotherapy treatment for both cell survival [110,111,112,113] and cell death [114,115,116].

### 5.1. Therapy Induced Autophagy

TMZ induces autophagy in glioma cells as demonstrated by the increase in LC3-GFP-positive vacuoles and levels of LC3B-II, as well as an accumulation of auto-fluorescent monodansylcadaverine in autophagic vacuoles [110,113,117]. Earlier studies demonstrated that clinically relevant doses of TMZ-induced autophagy without apoptosis [110]. These studies simultaneously showed that inhibition of autophagy through treatment with bafilomycin A, an inhibitor of vacuolar type H^+^-ATPase, led to caspase-3 activation and subsequent apoptosis, illustrating that autophagy is one mechanism by which glioma cells can escape cell death. Knizhnik *et al.* [113] showed that TMZ-induced autophagy occurs as a result of *O6-*MeG lesions that arise from TMZ treatment. Exogenous expression of the repair enzyme MGMT inhibited induction of autophagy in these glioma cultures while inhibition of MGMT led to an increase in autophagy. Disruption of the MSH2-MSH6 complex or ATM kinase, via siRNA knockdown, abrogated autophagy, demonstrating that an intact MMR and ATM kinase is required for autophagy induction. Time course studies following TMZ treatment showed that autophagy is detected as much as two days before apoptosis in several glioma lines. Inhibition of autophagy in these studies led to not only an increase in apoptosis and allowed apoptosis to occur at an earlier time point. Autophagy was also shown to precede and be required for senescence, thus explaining how autophagy could contribute to cell survival following TMZ treatment.

Autophagy in gliomas has also been shown to be stimulated not only through cellular damage, as seen with TMZ treatment, but also through various metabolic stresses, such as nutrient or growth factor deprivation, providing the cell with a survival mechanism. Filippi-Chiela *et al.* [118] focused their work on the effects of combination treatment of TMZ with Resveratrol, a dietary polyphenol known to inhibit proliferation. Glioma cell treatment with resveratrol and TMZ led to an increase in autophagy. Autophagy here had no role in the cytotoxicity of the treatment but rather acted as a cytoprotectant mechanism. Similarly, glioma cells treated with the EGFR tyrosine kinase inhibitor, erlotinib (Tarceva^®^), underwent autophagy with reduced cell death. Co-treatment with the autophagy inhibitor, chloroquine (CQ), and erlotinib increased cell death [119]. Therapy-resistant PTEN-mutant gliomas fail to undergo significant cell death in response to PI3K and mTOR inhibitors; however, treatment with a dual PI3K-mTOR inhibitor, PI-103, led to an induction of autophagy [111,120]. Apoptosis was increased by inhibiting autophagosome maturation, with bafilomycin A1, in conjunction with PI-103 treatment. Interestingly, this increase in apoptosis was not achieved with individual PI3K, Akt, or mTORC inhibitors, including rapamycin, in combination with bafilomycin A1; the combined inhibition of autophagy, mTOR and PI3K was required for cell death. These observations were extended using the PI3K-mTOR clinical inhibitor, NVP-BEZ235 (Novartis). Initial *in vivo* studies evaluating the therapeutic efficacy of NVP-BEZ235 alone showed an increase in survival of mice in an U87 intracranial model over vehicle-treated mice [121]. Another *in vivo* xenograft model showed that NVP-BEZ235 or CQ alone slowed tumor progression but tumor regression and increased apoptosis was only achieved when NVP-BEZ235 in combination with CQ was administered. This further supports the need to inhibit autophagy to drive tumor cells towards cell death and ultimately achieve tumor regression.

Inhibiting autophagy in combination with other therapies is a promising approach to reduce tumor cell survival following chemotherapy and is now being tested in the clinic. CQ continues to be evaluated as a treatment for gliomas [122] with clinical data indicating an increase in survival in patients in a phase II trial that added 150 mg daily dose of CQ as part of their adjuvant regimen [108]. A study by Sotelo *et al.* [123] showed patients in the CQ treatment arm had a median-survival of 24 months *versus* 11 months in the control group with a secondary study by Bricero *et al.* validating these results [124]. Another phase I/II active trial is evaluating the effects of adding hydroxychloroquine to temozolomide and radiation in newly diagnosed glioblastoma patients [108]. CQ is well tolerated for long periods of time with doses as high as 500 mg daily, making it a promising drug to be combined with the current standard of care [122]. However, further research is need into the safety of this regimen. Autophagy helps maintain homeostasis in many of the body’s organs and inhibiting autophagy may sensitize normal cells to chemotherapy [109]. Simultaneously, resistance to autophagy inhibition may occur some tumors rendering this approach non-applicable [125].

### 5.2. Radiosensitivity and Autophagy

Radiotherapy constitutes an important part of GBM treatment; however, obstacles remain in that cells resistant to radiation contribute to tumor recurrence. Therefore, it is important to elucidate the mechanisms responsible for differences in radiosensitivity of glioma cells. Autophagy is one of the mechanisms implicated in the response of glioma tumor cells to radiation. Several studies have shown that autophagy enhances radiosensitivity and leads to the induction of cell death [114,115]. Radiation alone or in combination with TMZ has been shown to activate autophagy in selected glioma cultures that are highly radiosensitive. Knockdown of key components of the autophagy pathway, Beclin-1 and Atg-5, inhibited autophagy, reducing sensitivity to radiation alone or in combination with TMZ. Sensitization of glioma cultures to radiation was achieved after treatment with rapamycin, a known inducer of autophagy [115]. Studies by Zhuang *et al.* on glioma-initiating cell lines observed similar results. CD133+ neurospheres showed increased autophagy when exposed to rapamycin and radiation. *In vivo* treatment of mice with intracranial tumors with rapamycin and radiation resulted in increased survival compared to radiation or rapamycin alone [114]. Similarly, glioma cells treated with the dual PI3K-mTOR inhibitor, NVP-BEZ235, exhibited greater sensitivity to IR as a result of the activation of autophagy [116].

In contrast, other studies have linked autophagy as a cytoprotective mechanism induced in response to IR and thus, subsequent inhibition has been linked to increased radiosensitivity. Radiotherapy of primary glioma stem-like cells with CQ alone or in combination with a PI3K/mTOR inhibitor increased cell death [112]. In the context of NVP-BEZ235, it was shown that inhibition of NVP-BEZ235-induced autophagy with 3-methyladenine or CQ increased radiosensitivity. One explanation proposed to explain the conflicting studies is that NVP-BEZ235 simultaneously induces autophagy (decreasing radiosensitivity) and impairs DNA damage repair (increasing radiosensitivity) [126]. The balance between autophagy and impairment of DNA damage repair may be a critical determinant of radiosensitivity.

It is important to note that radiosensitization by NVP-BEZ235 is dependent on the drug-irradiation schedule. Cells treated with NVP-BEZ235 prior to IR arrested in G1 and showed less DNA damage as assessed by histone γH_2_AX expression. Interestingly, cells in this schedule regimen had less DNA damage than irradiation only controls, potentially due to an induction of a survival mechanism such as autophagy. In contrast, NVP-BEZ235 administration before, during, and after radiation sensitized glioma cultures which was characterized by an increase in apoptosis, DNA damage, a prolonged G2/M arrest [127].

To summarize, the role of autophagy in resistance to therapy is unusually complex because autophagy can enhance cell death or survival, often depending on the cell identity and the details of the treatment. Additional laboratory studies and clinical trials are needed to determine whether autophagy can be manipulated to enhance cancer therapy.

## 6. Emerging Approaches to Therapies

### 6.1. Development of Novel TMZ-like Drugs

TMZ is a successful drug with oral administration, manageable side effects and enhanced survival for patients with glioblastomas [6,7,13]. However, its most toxic product, *O6-*MeG, is readily reversed by MGMT, and methylation of DNA at other sites is reversed by BER. A drug with less readily repaired products would enhance therapy in the clinic. However, TMZ may reach brain tumors and react with DNA more effectively than these new compounds. Fortunately, TMZ and related compounds have been extensively studied, and this information will facilitate design of TMZ-like drugs with increased anticancer activity and good pharmacokinetics.

**Figure 1:**
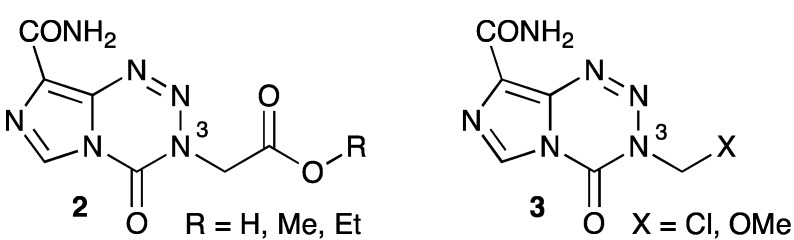
Novel TMZ-like drugs.

Two approaches are currently being taken to develop new TMZ derivatives that are resistant to, or avoid, the two principal constraints on the ability of a tumor to respond to TMZ therapy, viz, MGMT and MMR dependence. One approach has been to adjust the imidazotetrazine 3-substiuent so that the group transferred to DNA G-*O*6 sites is either not recognized or not repaired by MGMT. A range of neutral polar and charged G-*O*6 substituents resistant to cleavage by MGMT has been characterized [128]. Several such substituents have been incorporated into experimental imidazotetrazines **2**, **3** (Figure 1). Other than the free carboxylic acid (**2**, R = H), these compounds have all been shown active against GBM and colorectal cells lines that are resistant to TMZ, whether because of proficient MGMT or having deficiency or mutation in the MMR components hMLH1 or hMSH6. Onset of repair processes was slower than for TMZ and replication-independent (*i.e*., MMR-independent) DSBs were implicated in the cellular mechanism. The inactivity of the free carboxylic acid is interesting as it indicates a prodrug role for the esters in facilitating cellular penetration of the ionizable carboxylic functionality [129]. Carboxymethylguanine is a known mutagenic metabolite, resistant to MGMT repair but is a potential *O*6-MeG precursor that is generated from nitrosoglycine that forms during amino acid digestion in the stomach [130,131].

**Scheme 5:**
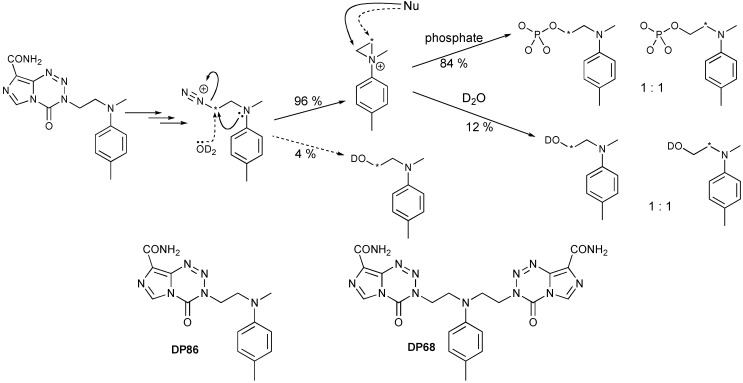
Reaction of DP86 in phosphate buffer pD = 7.4. * Sites of ^13^C labelling.

In the second approach a complete switch of chemical mechanism has been achieved with the dual aims of avoiding MGMT and MMR dependence and making the drug more efficient than TMZ by generating pharmacological activity from the major reaction site on DNA, G*N*7 (70% for TMZ), rather than the minor (5%) G-*O*6 site. This advance employs a neighboring group participation mechanism to control the behavior of the released alkyldiazonium ions, Scheme 5. This serves the dual functions of controlling reactivity, so giving the electrophile time to locate its reaction site on DNA, and delivering an alternative form of damage to DNA. Since the response of tumors to TMZ is determined by the interaction of DNA repair systems with modified DNA, altering the electrophile would necessarily alter the profile of tumor responses. In these respects, the potential of the imidazotetrazines as acid-stable precursors of aziridinium ions was explored as these are reactive intermediates of proven clinical utility, widely found in or generated by synthetic and natural product anti-tumor drugs, e.g., nitrogen mustards. The bifunctional agent DP68 and its analogous monofunctional form DP86 are currently under preclinical investigation.

The aqueous chemistry of diazonium ions is beset by problems of competing hydrolysis, elimination and re-arrangement reactions, which are reduced for aziridinium ions. In a ^13^C labeling study, DP86 was shown to be an efficient precursor of aziridinium ions (Scheme 5). At the stage of the diazonium ion, there was 96% conversion to the aziridinium form with only 4% direct hydrolysis. Products of further reaction had the labeled atom scrambled so that it appeared equally at both positions of the ethyl chain: confirming that they were entirely derived via the aziridinium route. Highly effective control of the diazonium ions had been achieved—in sharp contrast to other agents designed as precursors of aminoethyldiazonium ions [132].

The *in vitro* profiling of these compounds is very promising. In screening against A2780 (MMR+, MGMT+) and A278-cp70 (MMR−, MGMT+) cells in the presence and absence of PaTrin2 [36], monofunctional compounds such as DP86 were as potent as mitozolomide (the more potent but myelosuppressive 3-chloroethyl analogue of TMZ). The bifunctional agents were significantly more active than TMZ. MMR dependence was greatly reduced (from about 27-fold effect on IC_50_ for TMZ to 2–5-fold) and MGMT dependence effectively null. NCI screening data showed that the new compounds were not uniformly cytotoxic and confirmed the absence of correlation between activity and MMR and MGMT. Moreover, matrix COMPARE analysis showed that the new agents are pharmacologically distinct from standard agents that generate aziridinium or diazonium ions, that react at G-*N*7 or G-*O6*, or crosslink DNA such as nitrogen mustards, nitrosoureas and cisplatin. The chemosensitizing effect of these compounds is also independent of p53 [133]. DP68 has further been shown to effectively crosslink DNA in cells [R.M. Phillips, personal communication] and the biochemical response to the crosslinks is mediated through the ATR/FANCD2 pathway [52]. This finding is doubly significant as it shows that there is an escape pathway for healthy cells to survive damage by DP68, and also that a tumor with deficiency or mutation in the ATR/FANCD2 pathway (which includes BRCA1 and BRCA2) would be hypersensitive to this agent.

### 6.2. Drugs Directed Against Isocitrate Dehydrogenase

Using large-scale sequencing, several novel and exciting glioblastoma-associated mutations were identified [134]. They found that 50%–80% of low-grade gliomas carried mutations of isocitrate dehydrogenase 1 (IDH1) or isocitrate dehydrogenase 2 (IDH2). Later studies showed that 5% of primary glioblastomas and 60–90% of secondary glioblastomas express mutant IDH proteins [135,136]. In addition, many acute myeloid leukemias bear IDH mutations. Although a variety of other tumor types bear IDH mutations, the percentages of mutation-positive tumors are much less than for glioblastoma and acute myeloid leukemia. Only one IDH gene copy is mutated, and either IDH1 or IDH2, but not both, is mutated. These enzymes catalyze the oxidative decarboxylation of isocitrate, producing α-ketogutarate (α-KG) and regenerating NADPH as part of the tricarboxylic (TCA) cycle. IDH1 is present in the cytoplasm and peroxisomes; IDH2 is mitochondrial. For both enzymes, arginines in the catalytic pocket (IDH1 R132 and IDH2 R140 or R172) were mutated. The uniqueness of these mutations suggested a gain-of-function mutation, and a subsequent study demonstrated that these mutated IDH enzymes reduced α-KG to an oncometabolite, 2-hydroxyglutarate (2-HG) [137]. Overexpression of these mutated IDH enzymes induces histone and DNA hypermethylation and blocks cellular differentiation.

Although 2-HG was only recently discovered, several exciting targets have been identified that might drive cancer growth and progression [138]. One appealing model is that 2-HG, which accumulates to high levels in cells with IDH mutations, competitively inhibits α-KG-dependent enzymes. This competition is plausible since the structures of α-KG and 2-HG are quite similar. There are approximately 70 known and predicted human α-KG-dependent dioxygenases. In particular, the TET family of enzymes hydroxylates 5-MeG to generate 5-hydroxymethylcytosine, which is a step in DNA demethylation. 2-HG may also inhibit histone demethylases, which are known to act as tumor suppressors. Finally, 2-HG may inhibit the EglN family that hydroxylates proline residues on HIFα. By inhibiting this reaction, 2-HG allows accumulation of HIFα and increases tumor cell responses to hypoxia. These are all exciting cancer-relevant models, and undoubtedly additional targets will be discovered.

A natural question is whether IDHs are targets for therapy. Although IDH is universally expressed, the unique IDH mutations could be specifically targeted, lowering levels of 2-HG and hopefully retarding tumor growth. This is particularly appealing for low-grade gliomas for which there are few appealing treatment possibilities. In two recent studies, promising IDH inhibitors were described [139,140]. Both the IDH1 and IDH2 inhibitors showed marked preferences for the cancer-mutated IDH enzymes. Wang *et al.* [139] inhibited the mutated IDH2 enzyme in leukemia cells, slowing cell proliferation and inducing differentiation. Rohle *et al.* [140] used the IDH1 inhibitor to slow proliferation of glioblastoma cells, induce demethylation of histones and enhance astroglial differentiation. These results have exciting applications for the clinic. For example, a mutated IDH inhibitor with low toxicity might delay progression of low-grade to high-grade tumors.

## 7. Conclusions

GBMs are chemotherapy-resistant tumors with limited treatment options. The current standard of care enhances the OS of patients but does not cure or prevent recurrences. Understanding the mechanisms that generate resistance is essential to developing more effective chemotherapies. Many studies have demonstrated that DNA repair pathways, such as MGMT, BER and MMR, reverse chemotherapy-induced damage and mediate resistance in gliomas. Inhibition of MGMT continues to be the main therapeutic approach to overcome resistance in GBMs. CSCs contribute to tumor recurrence as once therapy is completed the cells can re-populate the tumor. Furthermore, it has been suggested that GBM CSCs can differentiate into GECs to provide the tumor with the vasculature necessary to survive. In addition, autophagy may facilitate survival of some cells following radiotherapy and chemotherapy making inhibition of autophagy a promising new target for therapy. However, autophagy can induce cell death, demonstrating that a better understanding into what dictates survival *versus* cell death roles of autophagy is still required.

Investigators are exploring a variety of novel approaches to improve GBM therapy. Currently, medicinal chemists are synthesizing new imidazotetrazine analogues that hopefully will be more effective than TMZ. The key to this approach is to circumvent DNA repair pathways with drugs that form adducts that cannot be processed. Furthermore, inhibitors that specifically target mutated IDH may provide physicians with a drug to slow or prevent the progression of low-grade tumors to GBMs with few side effects. Elucidation of the mechanisms that promote resistance and recurrence may provide novel targets that will improve the standard of care and overall survival.
